# Celebrating Transgender Day of Visibility 2023: an interview with Leland Graber and Ezra Kottler

**DOI:** 10.1038/s42003-023-04721-5

**Published:** 2023-03-31

## Abstract

March 31st marks Transgender Day of Visibility, an opportunity to celebrate and elevate the achievements of the transgender community. As part of our annual celebration of Transgender Day of Visibility, we reached out to Leland Graber and Dr. Ezra Kottler, two early-career transgender biologists who shared their own experiences and perspectives on improving support systems for the transgender research community.

**Figure:**
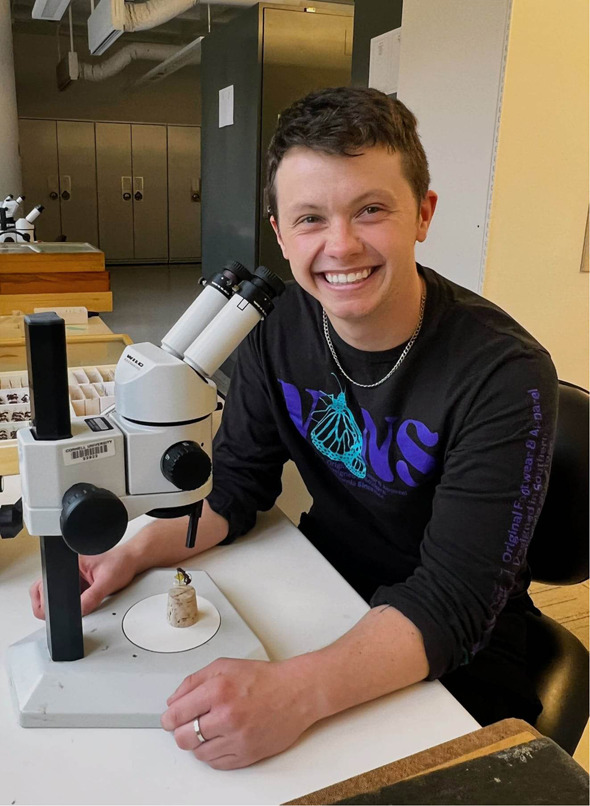
Leland Graber (he/him) is a PhD student in the Department of Entomology at Cornell University. Leland Graber.

**Figure:**
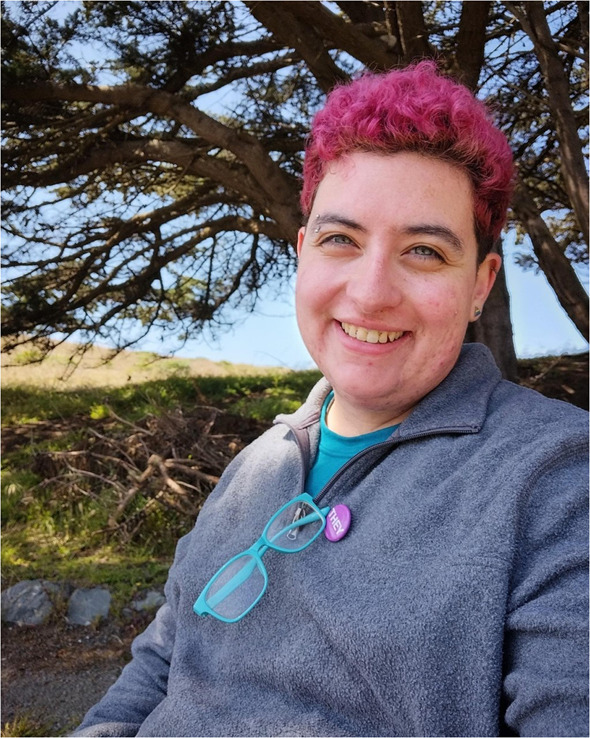
Dr. Ezra Kottler (they/them) is a postdoctoral researcher and Smith Conservation Fellow at the University of Colorado Boulder. Ezra Kottler.

Please tell us about your academic background and current research interests?

**Leland Graber (LG):** I got really into honey bees after writing a research paper on them in my freshman writing class at the University of Minnesota (UMN), so much so that I declared an entomology minor before taking a single entomology class. Between my undergraduate research at UMN, an internship, and a postbac fellowship, I had worked with honey bees, springtails, bed bugs, and mosquitoes, all before starting grad school! In my current graduate work at Cornell, I am studying the evolution of the harvester ant genus *Pogonomyrmex*. This genus is particularly interesting because many species eat seeds—a rare diet choice for ants. My work addresses how this uncommon appetite for seeds evolved to become so prevalent within the group.

**Ezra Kottler (EK):** My main area of study is investigating how wetland plants are responding to rapid environmental changes associated with climate change. I received my PhD in Biology from George Washington University last year for my dissertation looking at marsh grasses migrating inland with sea-level rise in the Chesapeake Bay. I am now a postdoctoral researcher at the University of Colorado Boulder studying the evolutionary resilience of two Californian endangered wetland plants to environmental extremes. I am working with conservation practitioners so that insights from my research can help inform ecological restoration efforts.

What originally inspired your passion for your field of research?

**LG:** I have always been a little obsessed with trying to figure out how animals, especially insects, evolved to become the way they are today. But because I don’t have a time machine and I cannot travel back hundreds of millions of years ago, the best I can do is phylogenetics (the study of evolutionary history and relationships between organisms). Technological advances of the past few decades have really expanded what phylogeneticists can do with DNA sequencing and computational analytics, so I feel lucky to be doing this work right now.

**EK:** In college I became fascinated with the complex web of interconnectedness between different living and nonliving members of an ecosystem. Climate change is altering ecosystems so rapidly that there are infinitely more questions than we have time to answer, and by the time we have there will be more! The urgency of this research is also very motivating as we work to conserve habitats that organisms and communities rely upon.

What would you consider to be your proudest research achievement so far?

**LG:** My favorite part of grad school has been mentoring many excellent undergraduate and junior grad students. Just last month, one of my first undergraduate mentees, Jenna Webb (now a grad student at University of Wisconsin), published her undergraduate work on the diversity of the bacterial symbiont *Wolbachia* in spiny ants. It was her first first-author paper! I’m really proud to be a co-author on that paper.

**EK:** I am honored to be a recipient of the highly competitive Smith Conservation Fellowship, which is funding my current postdoctoral research. I designed this research project from the ground up, and am working on understanding the resilience of the endangered plants I study from many angles. Right now I’m running experiments in the greenhouse and in created wetlands in California, I’m studying the genetics of these species, and I’m working with native plant growers and governmental partners to better understand the economic and logistic factors that challenge our ability to conserve and restore habitats effectively.

There is an increasing recognition of the need for more inclusivity and trainee resources in STEM, but are there any specific initiatives you would like academic institutions to support?

**LG:** With hundreds of anti-trans laws proposed in state legislatures this year–and thirteen already passed–it’s a pretty rough time to be trans in the US. I would like to see academic institutions (especially in states where gender-affirming care is threatened) more publicly defend trans rights. Much of the current anti-trans climate is being made worse by the spread of misinformation, sometimes even by major news outlets, so I would really like to see professors and academic scientists use their positions of high public trust to speak out against these misconceptions and outright transphobic lies.

**EK:** LGBTQ+ people in the U.S. are more likely to be affected by poverty and to experience housing insecurity (Williams Institute 2020; https://escholarship.org/uc/item/3cb5b8zj). In my experience of going through graduate school in a high cost of living city, grad students without familial financial support as a safety net can sometimes struggle to afford basic housing, food, and medical expenses. There needs to be more funding from academic institutions to specifically support grad students from minoritized groups, and it needs to be disbursed in methods other than reimbursement, which puts additional strain on grad student finances.

Are there ways your field could better support transgender researchers and improve diversity?

**EK:** There are a lot of unique safety concerns for transgender field scientists as they travel to and work in remote areas. Last year I founded the Trans & Gender-nonconforming Field Alliance, and together we are working to create resources for allies and for fellow transgender field scientists to support their safety and well-being in field research.

**LG:** In biology, specifically, I strongly believe that the ways we teach students about sex and gender, even at the K-12 level, can make a big difference in the public opinion of transgender people. Anti-trans pundits often claim that they are “defending biology” by making it difficult for trans people to access gender-affirming care. These beliefs seem rooted in the idea that biology is narrow and static, but really, the biological world is diverse, expansive, and constantly changing. If educators taught their students about the myriad of ways organisms reproduce instead of focusing on just one or two examples, I think it would have a lot of potential to change the ways people think about human sexual behavior and gender expression.

What advice would you give to other trainees who may be struggling with their identity or finding a support community?

**EK:** It can be incredibly hard to be the first out trans person in a workplace – I had this experience in both my department and the field station I worked at in grad school. In addition to support from local friends, joining LGBTQ + STEM affinity groups online really helped me to feel less isolated and more confident advocating for myself in the workplace. You can check out the resource page on the Field Alliance website for a list of LGBTQ + Affiliation Groups, or join our Alliance if you are a field scientist!

**LG:** When looking for mentors or advisors, be explicit in your search that you’re looking for an inclusive and supportive working environment. It can feel scary to disclose vulnerable parts of your identity to more powerful people in your field, especially if you have only just started to realize that you aren’t straight and/or cis. While you may encounter some disappointing reactions, it is important to hear that negativity outfront to avoid working with these people in the long run. Thankfully my experience in entomology has shown me that these bad apples are relatively rare; most of my colleagues are kind, supportive, and eager to learn. I continue to find that people often surprise me in very good ways.

What question do you wish people would ask you more often (and what would be your response)?

**EK:** The past few years have been really challenging for the LGBTQ + community as anti-LGBTQ + legislature and violence are escalating at an alarming rate. I have really appreciated the few occasions when colleagues have reached out to me about how I’m doing in the wake of one of these events (such as the Q Club shooting which occurred two hours South of where I live the evening before Transgender Day of Visibility). It tells me that they are staying informed about the news of these events that profoundly impact my community, and they care about me. My response is probably that I’m not okay. These events weigh on my mind heavily. The best I can do is give mutual support to my community and do my part by continuing to advocate for queer and transgender scientists.

**LG:** I love it when people ask me what my favorite insect is. I have a few favorites but I usually choose treehoppers because most people haven’t heard of them. I would highly encourage anyone who hasn’t to look up treehoppers on Google Images–you definitely will not regret it!

*This interview was conducted by Senior Editor, George Inglis*.

